# Baltic Sea ecosystem-based management under climate change: Synthesis and future challenges

**DOI:** 10.1007/s13280-015-0661-9

**Published:** 2015-05-28

**Authors:** Thorsten Blenckner, Henrik Österblom, Per Larsson, Agneta Andersson, Ragnar Elmgren

**Affiliations:** Stockholm Resilience Centre, Stockholm University, 106 91 Stockholm, Sweden; Institute of Biology and Environmental Science, Linnaeus University, 391 82 Kalmar, Sweden; Department of Ecology and Environmental Science, Umeå University, 901 87 Umeå, Sweden; Department of Ecology, Environment and Plant Sciences, Stockholm University, 106 91 Stockholm, Sweden

**Keywords:** Eutrophication, Persistent organic pollutants, Overfishing, Ecosystem-based management, Climate change

## Abstract

Ecosystem-based management (EBM) has emerged as the generally agreed strategy for managing ecosystems, with humans as integral parts of the managed system. Human activities have substantial effects on marine ecosystems, through overfishing, eutrophication, toxic pollution, habitat destruction, and climate change. It is important to advance the scientific knowledge of the cumulative, integrative, and interacting effects of these diverse activities, to support effective implementation of EBM. Based on contributions to this special issue of *AMBIO*, we synthesize the scientific findings into four components: pollution and legal frameworks, ecosystem processes, scale-dependent effects, and innovative tools and methods. We conclude with challenges for the future, and identify the next steps needed for successful implementation of EBM in general and specifically for the Baltic Sea.

## Introduction

Human activities have both directly and indirectly altered ecosystem dynamics worldwide, with significant and often negative environmental and economic consequences (Jackson et al. 2001; Halpern et al. 2008a; Conversi et al. 2014).

An adaptive governance or management strategy is required to manage ecosystems effectively (Folke et al. 2005). Ecosystem-based management (EBM) has emerged as the dominant strategy for managing ecosystems, with humans seen as parts of the system to be managed (Misund and Skjoldal 2005; Ruckelshaus et al. 2008; McLeod and Leslie 2009; Tallis et al. 2010; Berkes 2012). This approach differs from historical resource management by defining management strategies for whole ecosystems, rather than for individual components of the system (Leslie and McLeod 2007; Berkes 2012). Furthermore, EBM differs by considering interactions among ecosystem compartments and sectors (e.g., shipping and fishing), as well as the cumulative impact from resource use by different sectors of society (Rosenberg and McLeod 2005).

Human activities have substantial effects on marine ecosystems, such as overfishing, eutrophication, toxic pollution, habitat destruction, and climate change (Halpern et al. 2008a). It is important to advance the scientific knowledge of the cumulative and interacting effects of these diverse activities, in order to support effective implementation of EBM. This special issue of *AMBIO* is a contribution to this process and provides examples of scientific findings intended to improve our understanding of and capacity to manage the ecosystem of Baltic Sea.

This synthesis paper shows how the findings presented in other articles in this special issue contribute to EBM of the Baltic Sea environment. It is not a general or comprehensive review of scientific findings on the Baltic Sea, but instead aims to synthesize new scientific knowledge produced by the two strategic Swedish research programs “Ecosystem dynamics in the Baltic Sea in a changing climate perspective,” ECOCHANGE, a collaboration between Umeå and Linnaeus Universities, and “Baltic Ecosystem Adaptive Management,” BEAM, at Stockholm University. We focus on (a) pollution and legal frameworks, (b) ecosystem processes, (c) scale-dependent effects, and (d) innovative tools and methods. Finally, future challenges including ecosystem responses to projected climate change scenarios are identified to address the next steps needed for successful implementation of EBM in general, and specifically in the Baltic Sea. In the following, all citations that refer to papers of this special *AMBIO* issue are marked in bold.

## Pollution and legal frameworks

Coastal marine systems, estuaries, and regional seas in Europe are heavily impacted by nutrients loads from land, often leading to eutrophication symptoms such as harmful phytoplankton blooms and anoxic bottom waters (Anderson et al. 2002; Stal et al. 2003; Kemp et al. 2005; Diaz and Rosenberg 2008; Conley et al 2011). The nutrient load to the Baltic Sea increased steadily until the late 1980s, and still causes large cyanobacteria blooms in summer (Kahru and Elmgren 2014), extensive anoxic areas, both shallow and deep (Carstensen et al. 2014), and mass mortality of zoobenthos, reducing food availability for demersal fish, such as the commercially important cod (*Gadus morhua*) (Karlson et al. 2002).

To reduce the pollution to the Baltic Sea, the Helsinki Commission (HELCOM) in 2007 adopted the Baltic Sea Action Plan (BSAP) (HELCOM 2007, revised in HELCOM 2013) within the framework of the Helsinki Convention for the Protection of the Baltic Sea Environment. This plan relies mainly on two legal instruments, namely the EU Water Framework Directive (WFD) and the EU Marine Strategy Framework Directive (MSFD). These legal instruments require that the coastal states of the Baltic Sea that are EU members (all but Russia) implement and enforce legal measures to abate eutrophication, mainly through rules aiming to minimize the release of discharges to coastal areas and eventually to marine waters, using an ecosystem-based approach. **Nilsson and Bohman** (2015) analyzed the role of law in the management of Baltic Sea eutrophication and the legal instruments used to implement an ecosystem-based approach. Their principal conclusion is that to properly enforce ecosystem-based adaptive management, the management structures and tools need to be further developed, for example, through clarification of duties and responsibilities for their realization and by proposing more concrete management measures, such as farm-specific nutrient regulations.

In addition to nutrients, the Baltic Sea has also been severely polluted by persistent organic contaminants, such as PCDD/Fs, PCBs, HCHs, HCB, and DDTs. From at least the 1960s, elevated concentrations of these contaminants caused severe adverse effects on Baltic Sea biota, and for example, grey seal (*Halichoerus grypus*) and white-tailed eagle (*Haliaeetus albicilla*) were almost driven to extinction in the region. Significant decreases have since late 1970s/early 1980s been observed for most of these pollutants (**Nyberg et al.**^2015^), leading to significant recoveries of affected mammals and birds (Helander et al. 2008; Roos et al. 2012). However, concentrations of dioxin-like compounds in fish are still higher in the Baltic Sea than in for instance the North Sea. Some contaminants (CB-118, chlorinated dioxins and DDE) still exceed the suggested target levels at some sites and in some monitored Baltic Sea species. This suggests that concentrations may still be too high to fully protect the most sensitive organisms (**Nyberg et al.**^2015^), including humans, and hence diet recommendations are still needed. Dioxins and dioxin-like PCBs in fatty fish sometimes exceed the maximum limits for human consumption and animal feed established by the European Commission (Assefa et al. 2014). Natural brominated substances produced by algae may augment stresses from such anthropogenic compounds (Haglund et al. 2007; Löfstrand et al. 2010; **Bidleman et al.**^2015^). Overall, the management of organic contaminants is a major success story in Baltic environmental governance (**Elmgren et al.**^2015^), but for some specific contaminants further action is needed, e.g., for dioxins and several endocrine-disrupting chemicals (UNEP/WHO 2013).

## Ecosystem processes

This special issue provides several examples of new insights into Baltic Sea ecosystem processes. For example, the new metagenomic sequencing methods combined with potent bioinformatics instruments (Dupont et al. 2014) are now rapidly being applied in ecosystem-based research. This approach is fundamentally improving our knowledge of the identity of the microorganisms, most important in driving major global nutrient cycling in aquatic ecosystems. In the Baltic Sea, a strong link between environmental conditions and the composition of the microbial community exists (**Ininbergs et al.**^2015^; **Lindh et al.**^2015^). This indicates that changes in salinity may lead to rapid changes in the bacterial community, with implications for food-web functioning, contaminant breakdown, and biogeochemical cycling. Likewise, **Legrand et al.** (2015) show that changes in bacterial as well as phytoplankton composition and production are related to hydrographic conditions. They found that bacteria responded proportionally to increased temperature, and that both heterotrophic bacteria and small flagellates contributed significantly to the total carbon production. These studies show the importance of including microorganisms (including viruses) in pelagic food-web models.

Every summer large blooms of filamentous cyanobacteria characterize surface waters of the Baltic Sea (Wasmund 1997). Recent research indicates that such blooms today occur almost 3 weeks earlier than 35 years ago (Kahru and Elmgren 2014). These bloom-forming cyanobacteria fix dissolved nitrogen gas, adding large amounts of bioavailable nitrogen to the ecosystem (Larsson et al. 2001; Stal et al. 2003; Voss et al. 2005; Degerholm et al. 2008). This fixed nitrogen is incorporated in food webs via two major pathways:(1) by direct metazooplankton grazing on cyanobacteria and (2) through uptake of the exuded nitrogen by other primary producers that are further grazed in both microbial loop and classic food chains (**Andersson et al.**^2015^; **Karlson et al.**^2015^). The smallest phytoplankton, picoplankton, are very efficient in using such exudates (Ploug et al. 2011). These small primary producers are directly grazed by metazooplankton to a greater extent than previously realized, thus bypassing the microbial loop and contributing effectively to secondary production (Motwani and Gorokhova 2013; Majaneva et al. 2014). Therefore, fixed nitrogen originating from cyanobacterial blooms contributes to production of both zooplankton (Hogfors et al. 2014; **Karlson et al.**^2015^) and benthos (Karlson et al. 2014), and plays a crucial role for maintaining good feeding conditions for larvae and young-of-the-year fish in summer, the period of recruitment and the highest nutritional needs.

In general, shallow coastal areas are important spawning and feeding grounds for many organisms, including juvenile fish. New results show that predation by sticklebacks (*Gasterosteus aculeatus*) in coastal areas of the Baltic Sea can strongly reduce survival of larval perch (*Percafluviatilis*), but that this effect wanes rapidly as the perch grow, likely due to stickleback gape limitations and digestion constraints (**Byström et al.**^2015^). These results suggest that persistence of coastal piscivore populations is likely to be dependent on the availability of recruitment habitats, where early interactions with temporarily high densities of sticklebacks can be avoided. For another Baltic coastal fish of freshwater origin, the northern pike (*Esox lucius*), sub-populations appear to be locally adapted to their freshwater recruitment environments, an important finding for the management of such species, allowing wetlands to be managed to strongly promote spawning and recruitment success (**Larsson et al.**^2015^).

The commercially most important fish in the Baltic Sea is cod (*Gadus morhua*). The cod stock reached high biomasses in the early 1980s, but collapsed in the late 1980s due to overfishing and low recruitment success. This led to a severe ecosystem-wide regime shift (Casini et al. 2009; Möllmann et al. 2009). However, a slight recovery of the stock has now been reported (Eero et al. 2012), but the causes and mechanisms still remain controversial (Cardinale and Svedäng 2011; Möllmann et al. 2011; Svedäng and Hornborg 2014). A statistical food-web model (Blenckner et al. 2015a) indicated that complete recovery of this severely altered ecosystem is unlikely under current temperature and salinity conditions. The ecosystem is more likely to regenerate toward an ecological baseline with lower, more variable cod biomass, even under very low exploitation pressure, with severe economic consequences likely for the fishery (Blenckner et al. 2015a). This is of particular importance as management of depleted fish stocks has traditionally been treated as a management of single species, related to the level of exploitation (Worm et al. 2009). It is therefore most important to gain an understanding of the dynamics of commercially exploited fish stocks in an ecosystem context, including the effects of multiple drivers on the food web.

As Blenckner et al. (2015a) show, drivers of ecological processes can have synergistic effects leading to complex ecosystem responses. An example of such synergistic interactions, is the experimental study of Vehmaa et al. (2013) showing combined effects of temperature and acidification on zooplankton responses to toxic cyanobacteria. Under these multiple stressors, naupliar development was promoted by the cyanobacteria, partly alleviating the otherwise negative effects of increased temperature and lower pH on zooplankton recruitment.

Dissolved organic matter (DOM) is a major chemical constituent of rivers flowing into the northern Baltic Sea, and its concentration has increased in recent decades (Erlandsson et al. 2008). A DOM increase is known to have two effects in coastal areas; optically, it changes the light climate and heats near-surface waters, and, as an energy source, it stimulates bacterioplankton production (**Andersson et al.**^2015^). DOM is also important by binding organic contaminants, thereby influencing their transport and fate processes **(Bidleman et al.**^2015^). In the future, higher levels of rainfall are projected to result in further increased riverine export of DOM, especially to the northern basins of the Baltic Sea (Reader et al. 2014). Mesocosm experiments, in which both DOM concentration and temperature were increased, also indicated considerable and differential responses in bacterial populations to synergistic climate change effects. This emphasizes the risk of inducing shifts in ecosystem function and carbon cycling in the future Baltic Sea (**Lindh et al.**^2015^). Overall, these data suggest that understanding synergistic effects of multiple drivers on ecosystem functioning is important for future management actions (Halpern et al. 2008b). This has been shown to be the case also in other semi-enclosed seas, such as the Mediterranean and Black Seas (Llope et al. 2011), and the North Atlantic (Holt et al. 2014).

## Scale-dependent effects

Human actions influence ecosystem dynamics and processes at multiple scales, both directly and indirectly, by changing socioeconomic conditions, such as global wheat and fish prices (Crona et al. 2015). The extent to which humans dominate the biosphere has increased at unprecedented rates already in the previous century, altering the dynamics of ecosystems throughout the world (Estes et al. 2011; Frank et al. 2011). In this context, **Borgström et al.** (2015) found that goals and measures in EBM are often not defined on a scale aligned with the scale of EBM target areas (Folke et al. 2007).

Ignoring the scales of interactions can hamper understanding of the functional dynamics of ecosystems. This will limit modeling capacity and prevent a theory-based anticipation of surprises, such as threshold effects (Cash et al. 2006; Griffith and Fulton 2014). Furthermore, management strategies are implemented at multiple and interconnected governance levels (Cash et al. 2006). These may vary from international EU fisheries or global conservation treaties down to regional and local marine-protected areas. All management strategies face problems with compatibility of scales in social-ecological systems, and it is therefore important to include scale-dependent ecological and governance processes in system analysis (Cumming et al. 2006).

The importance of defining scales in research related to the Baltic Sea is obvious, as this water body is one of the largest brackish water areas on Earth. It shows marked gradients in abiotic conditions, particularly salinity and temperature, as well as in human use, such as fishing and land-use in the catchment area. Loads of nutrients and DOM also vary greatly spatially (**Andersson et al.**^2015^), as do specific contaminants in biota (**Nyberg et al.**^2015^). This spatial scale dependence also affects Baltic Sea food-web structures and interactions. For example, large variations in spatial and temporal patterns of stickleback migration into perch spawning sites have been observed. Whether or not coastal perch populations will decline in response to increasing stickleback densities may be determined by the availability of spatial refuges for spawning, to which sticklebacks do not migrate or arrive late in the perch reproduction period (**Byström et al.**^2015^).

Coastal fish stocks have decreased in many areas of the Baltic Sea (**Larsson et al.**^2015^). The reasons for this decline are not well known but proposed explanations include severe coastal eutrophication affecting reproduction, food-web changes, including increase of sticklebacks, local overfishing, reduction by ditching of coastal wetlands used as spawning areas, and blocked migration routes. Restoring wetlands or creating new ones, and opening blocked migration routes may enhance reproduction of coastal fish species of freshwater origin (**Larsson et al.**^2015^). Such measures require good ecological knowledge, as for the northern pike, where the same local coastal area may be inhabited by several genetically distinct populations (**Larsson et al.**^2015^). These have evolved by natal homing to different spawning areas (wetlands) and individual fish may be adapted to different specific environmental cues.

The distribution range of the Eastern Baltic cod population has decreased progressively after the cod boom in the mid-1980s, and the stock today is concentrated in the south-western Baltic Proper, where it still finds suitable conditions for reproduction (Cardinale and Svedäng 2011). Simultaneously, the distribution of sprat (*Sprattus sprattus*), the main prey for adult cod, has shifted toward the NE Baltic Proper, where predation mortality has plummeted after the cod stock collapse (Casini et al. 2012). This is a clear illustration of the importance of including spatial scales for better understanding species interactions.

## Innovative tools and methods

New and innovative tools can help in developing the understanding of ecosystem processes, and improve ecosystem monitoring techniques as well as management of marine resources. Some recent examples, elaborated within the ECOCHANGE and BEAM programmes, are presented in this special issue.

Potential new methods for identifying long neglected microbial communities (‘the unseen majority’) have in recent decades revealed their ecological importance in ecosystem processes, also in the Baltic Sea (**Ininbergs et al.**^2015^; **Lindh et al.**^2015^). This approach is based on representative sampling followed by high throughput sequencing (HTC) of the vast array of unknown microbes, including viruses. This has led to the identification of major biotic and abiotic drivers of biogeochemical cycles in the Baltic Sea (Dupont et al. 2014; Larsson et al. 2014), and now starts to provide data for a better holistic understanding and management of the Baltic Sea, related to incipient harmful microbial blooms, human pathogens, vitamin producers, invasive species, etc. Due to its efficiency, this approach is likely to gradually replace simpler methods currently used in environmental monitoring, such as DNA barcoding (Gorokhova et al. 2013; Majaneva et al. 2014). Important new methods for understanding trophic interactions are molecular and chemical diet analysis (Motwani and Gorokhova 2013), isotope niche analysis (Karlson et al. 2014), and nanometre scale secondary ion mass spectrometer techniques (Nano-SIMS; see for example Ploug et al. 2011). Development of new biomarkers and bioindicators is important for assessing the effects of environmental stressors on Baltic Sea biota (Vehmaa et al. 2013; Hogfors et al. 2014). Many of these new tools have potential for future use in Baltic Sea monitoring as indicators for assessing biological effects of contaminants and other stressors, and for classifying environmental status.

**Undeman et al.** (2015) have developed a modeling tool for the Baltic Sea that simulates interactions between climate forcing, hydrodynamic conditions and water exchange as well as between biogeochemical cycling and organic contaminant transport and fate. The new model is integrated with the NEST modeling system used by HELCOM for decision support (www.balticnest.org/nest), and simultaneously represents dominant biogeochemical processes and addresses multiple stressors like pollution, climate change, eutrophication, and overfishing. It can be used to improve the management of contaminants, for example, to compare the efficiency of alternative emission reduction measures, the sensitivities of the different basins to pollution, and for optimizing monitoring programs.

It is also important to develop tools and frameworks that can assess the relative success of the EBM process. In recent years, the number of publications on EBM has increased rapidly, but there are few systematic, critical appraisals of EBM that integrates both ecological and socioeconomic aspects. **Borgström et al.** (2015) have developed an interdisciplinary, analytical framework that gives a high-resolution, systematic assessment of the degree of specificity, and integration of ecosystem aspects in EBM. They used this framework to evaluate five coastal EBM initiatives in Sweden and conclude that their framework provides a basis for a refined analysis of *how* to improve EBM in any given case. This requires turning understanding of the system into coherent, integrated and specified goals, measures, and monitoring/evaluation activities (**Borgström et al.**^2015^).

## Future challenges

The changing climate is a major current and future challenge. Projected future climate change varies across the Baltic Sea and its catchment, with the largest sea surface water (SST) changes expected in summer in the north (Bothnian Sea and Bothnian Bay) and in spring in the Gulf of Finland (**Andersson et al.**^2015^). In contrast, projected decreases in sea surface salinity (SSS) are largest in absolute value in the southern regions (Danish Strait region), whereas normalized values (as a fraction of present values) indicate the largest salinity decrease in the north. The decrease in salinity will mainly be caused by changes in runoff from land as a result of increased precipitation in the region. This is, however, a highly uncertain aspect of future projections, due to large variation in spatial rainfall patterns between climate models (Meier et al. 2012). The future transports of nutrients and organic pollutants from land to the Baltic Sea are also influenced by water runoff from land, which is influenced both by changes in climate (**Bring et al.**^2015^) and in land-use. Reliable hydrological transport models are therefore needed to provide scenarios of climate-induced changes in nutrient loads from surrounding countries (**Bring et al.**^2015^).

The projected future increase in freshwater runoff is likely to enhance transport of DOM, an important substrate for heterotrophic bacterioplankton. This may lead to an increase in the bacterioplankton:phytoplankton ratio (**Andersson et al.**^2015^). Wikner and Andersson (2012) showed that years with higher than normal runoff led to decreased production by phytoplankton but not by bacterioplankton in the northern Baltic Sea. A study by **Harvey et al.** (2015) shows that the coupling between light-absorbing colored dissolved organic matter (CDOM) and dissolved organic carbon (DOC) is not coherent in the Baltic. Different areas of the Baltic (both offshore and coastal) have clearly different CDOM pools and hence optical properties, which affect the reliability with which phytoplankton biomass as chlorophyll can be estimated through remote sensing (**Harvey et al.**^2015^).

In the Baltic Proper, future increased nutrient loads and higher temperatures are likely to enhance internal nutrient cycling (Meier et al. 2011), which may lead to an increase in primary production and deep-water oxygen consumption (**Andersson et al.**^2015^). Therefore, unless the nutrient load is decreased further, the volume of water and area of bottom affected by hypoxia/anoxia may increase (Meier et al. 2012). Higher temperature and decreased salinity may also, directly and indirectly, stimulate growth of bloom-forming cyanobacteria and augment the levels of cellular toxicity, through a synergetic interaction with eutrophication (El-Shehawy et al. 2012). An increase in nutrient availability may increase the risk of filamentous algal mats (“drift algae”) in coastal zones (Arroyo et al 2012).

Climate change will also affect the transport and fate of organic contaminants. Loss of ice cover will increase the surface area and time available for air-sea gas exchange. Increased precipitation will mean greater atmospheric deposition on the Baltic and its drainage basin. The delivery of contaminants from land to sea will be increased by greater runoff and discharge of DOM, which binds organic contaminants and may increase their mobility (**Bidleman et al.**^2015^).

Local change can be a result of altered global social and economic dynamics. Hierarchical theory suggests that an up-scaling hierarchy exists where local scales affect the regional scale and subsequently the global scale. But an inverse scale hierarchy also exists, where global scale dynamics may determine local scale dynamics (i.e., down-scaling; Peters et al. 2008). An example of the inverse scale hierarchy is when agriculture or fisheries are influenced by changes in economy and markets, technological advances, and institutional frameworks (Berkes et al. 2006). The influences of global dynamics, such as trade flows, on regional and local scales have increased over time (Folke et al. 2011) and this will certainly create challenges for EBM of many marine areas. Notably, future scenario projections (Hägg et al. 2014) conclude that changes in meat consumption and populations are potentially more important than climate effects for future nutrient runoff from the Baltic Sea catchment. Taken together this suggests that lifestyle changes will be relatively more important in the southern regions of the Baltic Sea drainage basin, while climate change will be more important in the north (Hägg et al. 2014).

How changes in climate, land-use, trade flow, human population, and life style will affect the Baltic Sea ecosystem in a cumulative and potentially synergistic way is still largely unknown and may pose a risk of sudden changes in ecosystem structure and function, i.e., the so called ‘regime shifts’ (Conversi et al. 2014; Blenckner et al. 2015a). Such risks need to be explored and predicted more specifically (Blenckner et al. 2015b; **Elmgren et al.**^2015^), also in relation to the EU Marine Strategy Framework Directive (MSFD). Also systemic delays in time exist in the Baltic Sea, in policy, implementation, ecosystem effects, and their detection by monitoring (Varjopuro et al. 2014). In policy and governance, it may take years from problem identification to decision and further to implementation (Elmgren 2001). Delays in ecosystem response are often caused by feedbacks that keep the ecosystem in the current state (Nyström et al. 2012). Therefore, improved cooperation between in-depth ecosystem research, social institutional science, modeling and management (Österblom et al. 2013; **Elmgren et al.**^2015^), comparative analysis between analogous case studies (Sandström et al. 2015; Valman et al. 2015) and scientific cooperation across geographical scales (Paasche et al. 2015) could improve the prospects for providing a solid transdisciplinary basis for science-based EBM. Such analysis could identify barriers associated with implementing an ecosystem approach, including not only challenges associated with coordination between sectors, but also experiences with how such barriers have been overcome in other regions or at other scales.

Further, long-term monitoring, including remote sensing is crucial (Ferreira et al. 2011; **Harvey et al.**^2015^) and should be maintained and enhanced by addition of automatic measuring stations/boys equipped with advanced sensor technologies for monitoring at all organismal scales. These data should then be combined in an integrated ecosystem assessment (IEA), which is a formal synthesis and quantitative analysis of information on relevant natural and socioeconomic factors, in relation to specified ecosystem management objectives (Levin et al. 2009). IEA involves scientists, public, stakeholders, resource managers, and policy makers, in formal evaluation processes that contribute to achieving the goals of EBM (Levin et al. 2009). Such integration of information is necessary to prepare EBM for the future, both in general and specifically in the Baltic Sea area.
